# Cognitive networks detect structural patterns and emotional complexity in suicide notes

**DOI:** 10.3389/fpsyg.2022.917630

**Published:** 2022-12-08

**Authors:** Massimo Stella, Trevor J. Swanson, Ying Li, Thomas T. Hills, Andreia S. Teixeira

**Affiliations:** ^1^CogNosco Lab, Department of Computer Science, University of Exeter, Exeter, United Kingdom; ^2^Department of Psychology, University of Kansas, Lawrence, KS, United States; ^3^Max Planck Institute for Human Development, Berlin, Germany; ^4^Institute of Psychology, Chinese Academy of Sciences, Beijing, China; ^5^Department of Psychology, University of Warwick, Coventry, United Kingdom; ^6^LASIGE, Departamento de Informática, Faculdade de Ciências, Universidade de Lisboa, Lisbon, Portugal; ^7^INESC-ID, Lisbon, Portugal

**Keywords:** data science, psycholinguistics, complex networks, text analysis, emotional profiling, cognitive network science

## Abstract

Communicating one's mindset means transmitting complex relationships between concepts and emotions. Using network science and word co-occurrences, we reconstruct conceptual associations as communicated in 139 genuine suicide notes, i.e., notes left by individuals who took their lives. We find that, despite their negative context, suicide notes are surprisingly positively valenced. Through emotional profiling, their ending statements are found to be markedly more emotional than their main body: The ending sentences in suicide notes elicit deeper fear/sadness but also stronger joy/trust and anticipation than the main body. Furthermore, by using data from the Emotional Recall Task, we model emotional transitions within these notes as co-occurrence networks and compare their structure against emotional recalls from mentally healthy individuals. Supported by psychological literature, we introduce emotional complexity as an affective analog of structural balance theory, measuring how elementary cycles (closed triads) of emotion co-occurrences mix positive, negative and neutral states in narratives and recollections. At the group level, authors of suicide narratives display a higher complexity than healthy individuals, i.e., lower levels of coherently valenced emotional states in triads. An entropy measure identified a similar tendency for suicide notes to shift more frequently between contrasting emotional states. Both the groups of authors of suicide notes and healthy individuals exhibit less complexity than random expectation. Our results demonstrate that suicide notes possess highly structured and contrastive narratives of emotions, more complex than expected by null models and healthy populations.

## 1. Introduction

Suicide is the outcome of complex, distressed emotional processing. It represents an extreme behavior that often arises from negative perceptions and distorted mindsets about the self, others and life itself (Rizk et al., 2019; Oquendo et al., 2020). Despite recent progress in using Big Data and natural language processing for the automatic detection and discrimination of suicide notes from other types of digital texts (Schoene and Dethlefs, 2016; Bayram et al., 2022), more work is required to understand how people who completed suicide organize and express emotions in their final writings (Palmier-Claus et al., 2012; Hallensleben et al., 2019). This challenging task requires the adoption of interpretable algorithms (as opposed to “black box" models such as deep neutral networks) that afford a greater understanding of the mental patterns and ways of thinking expressed in the final notes of those who completed suicide.

In this study, we used network science to uncover the emotional structure of suicide notes. Building on previous approaches using natural language processing to study the contents and structure of suicide notes (Teixeira et al., 2021), we described how emotions are recalled, organized, and distributed within such narratives. Specifically, our data-informed approach allows us to (1) quantify emotional connotations for each word and track which part of a suicide note is most emotionally-loaded, and (2) introduce a metric of *emotional complexity* to capture the tendency of a narrative to switch between positive, neutral and negative affective states.

Our methodology utilizes techniques from graph theory and network science (Newman, 2018), a field that lies at the intersection of psychology, linguistics, natural language processing and cognitive science (De Deyne et al., 2016; Stella et al., 2017, 2019; Siew et al., 2019; Lynn et al., 2020; Mehler et al., 2020; Lydon-Staley et al., 2021). Network science provides a set of quantitative tools to represent cognitive systems. Specifically, nodes often represent conceptual entities of interest (e.g., words/concepts/ideas) and edges indicate certain types of relationships between nodes (e.g., mental associations, morphological similarity). An accumulating body of research has shown that network models can reveal important structural properties of various cognitive systems (De Deyne et al., 2016; Siew et al., 2019). Moreover, network science has been applied to model processes that operate within those systems, such as the structure of writing styles (Amancio, 2015), properties of information search (Lynn et al., 2020), and the development of mental disorders (Borsboom, 2017). Representing text as a network, where nodes represent words that are connected according to co-occurrence relationships, has led to important insights such as identifying opposing factions in political debates (Corrêa et al., 2019), detecting distrust toward science in student populations (Stella et al., 2019) and highlighting online stances in social media (Stella et al., 2018; Stella, 2022). Our network approach improves the current practice of emotional profiling by taking one step forward from the “bag-of-words" model (i.e., treating a note as a mere collection of disconnected words). By connecting words that appear next to each other in the text, the resulting network offers additional insights into the mind of the writers. By reconstructing how words were associated together, it is possible to use network measures to identify key or peripheral concepts in the narratives of suicide notes (Teixeira et al., 2021). We adopt this approach here to perform a comparison between different parts of suicide notes, focusing on the last few words adopted by authors to bid farewell to their loved ones. Are these “last words" more central than others mentioned in the main body of suicide notes? Which emotions corresponded to these farewells?

People use words to express their emotions, with or without explicit reference to emotion terms. For instance, “thank you for supporting my work" expresses the feeling of gratitude without explicit usage of that emotion word. The task of detecting various kinds of emotions in text is also known as *emotional profiling*, which recently met an unprecedented flourishing thanks to the increasing availability of large-scale datasets describing cognitive features of words as represented by the human mind (Fellbaum, 2012; Mohammad and Turney, 2013; Boyd, 2017; Siew et al., 2019; Li et al., 2020b).

Previous studies have shown that valence (pleasure-displeasure) is one of the underlying dimensions of word meaning (Osgood et al., 1957). For instance, people tend to associate the word *freedom* with pleasant feelings, and *inequality* with unpleasant feelings. Mohammad (2018) conducted a study wherein participants were asked to rate the valence of over 20,000 English words, and found that valence ratings were highly consistent across raters. The averaged valence ratings of books and newspaper articles have been found to predict feelings and opinions in everyday life, such as prejudice against U.S minority groups (Li and Hills, 2021) and historical national well-being (Hills et al., 2019).

Emotions, however, are far more complex than pleasantness/unpleasantness (Plutchik, 1980). For example, anger, fear, boredom, and sadness are all negatively-valenced emotions, but each of them corresponds to different psychological and behavioral consequences. A more fine-grained approach to emotional profiling is to detect *specific emotions* (instead of simply valence and other properties) from the text. A common approach relies on the NRC Emotion Lexicon (Mohammad and Turney, 2013)—a large dataset that describes mental associations between 14,000 words and 8 frequently-experienced emotions (joy, trust, fear, surprise, sadness, disgust, anger, anticipation). For example, the word “pandemic" would elicit fear.

### 1.1. Connecting suicide notes to suicidal minds

The relevant literature on suicidal minds provides interesting theoretical insights that correspond with a data-driven investigation of emotional profiling of suicide notes. Existing theories on suicide indicate that although the prolonged feeling of depressed mood can increase the likelihood of suicide, it is insufficient to account for the act of suicide (Li et al., 2014). Therefore, negative emotions are expected to be found in suicide notes but they cannot represent a coherent and comprehensive picture of the complex landscape that brought individuals to end their lives. A more articulated framework that might support the appearance of suicidal thoughts is psychological pain theory, or “psychache,” introduced by Shneidman (1999) and extensively reviewed within clinical psychology (cf. Xie et al., 2014).

Psychological pain is a negative introspective experience that is positively associated with suicide risk, independent of depression. The psychological pain theory argues that suicide is not just a drive toward death, but an escape from unbearable pain. The motivation to avoid pain is especially dangerous for depressed individuals because depression is often associated with decreased motivation to experience pleasure (Xie et al., 2014) as well as heightened sensitivity to anxiety (Demirkol et al., 2022). Although it is difficult to identify causal relationships between negative emotional states and clinical suicide ideation, extensive literature indicates that prolonged psychache can alter the mental schemas with which individuals perceive their surroundings and the prospects of recurring to suicide. A key reason behind such change is the perception of psychache being unbearable and the willingness to avoid such psychological pain through a suicidal act (Shneidman, 1999; Li et al., 2014). Associating suicide with the relief from the prolonged psychological pain, people who took their lives may have attributed positive feelings toward the their last decision. This positive feeling may have been reflected in their last letters, especially in the last sentence they said to this world. In study 1 we tested this hypothesis.

As noted by Shneidman (2004), a pioneering psychologist in suicide research, “suicide notes might prove to be the royal road to the understanding of suicide phenomena.” Echoing this view, plenty research has been conducted to describe how suicide notes are different from other language. One common approach is LIWC (Linguistic Enquiry and Word Count), a text analysis tool invented by Pennebaker (2015). LIWC contains over 50 dimensions of linguistic features (e.g., negative emotions and pronouns). It searches through text and count their presence. For example, the sentence “I love you” would receive 33.3% on positive emotion dimension because one of the three words is a positive emotion. LIWC has been used to explore suicidal minds. For example, Pennebaker et al. found suicidal poets use substantially more negative words (Baddeley et al., 2011) and words pertaining to the individual self (Stirman and Pennebaker, 2001). The current study takes a more fine-grained network approach to explore the emotional complexity in the letters as well as the emotional content that is absent. Emotional complexity, sometimes referred to as emotion dialecticism (Bagozzi et al., 1999; Scollon et al., 2005) or core affect variability (Kuppens et al., 2007), captures differences in the way pleasant or unpleasant emotions are experienced. Evidence for emotional complexity has been accumulating in various research paradigms, such as recalling prior experience, judging hypothetical scenarios, and summarizing experience across a period of time (Barrett, 1997; Robinson and Clore, 2002). The psychological pain theory of suicide (Shneidman, 1999) suggests that suicide ideation is characterized by a complex set of negative emotions as well as a positive perception of suicide as a solution to pain avoidance. Therefore, it is possible that suicide notes may entail higher level of emotional complexity (a mixture of positive and negative emotions) compared to language produced by healthy individuals. We test this hypothesis in Study 2. Please notice that our aim is to reconstruct a network of words/emotions from group-level psychological data, assessing their statistical significance with quantitative methods. We thus focus on group-level comparisons, with the limitation that our results cannot be misconstrued to individual-level analyses. To better investigate this aspect, we also perform individual-level triad counts to test whether the conflict detected at the group level is found also in differences in distributions coming from individual-level responses.

### 1.2. Manuscript outline

This manuscript has the following structure. The Section 2 outlines the construction of the cognitive network representing the selected corpus, as well as the cognitive datasets and statistical methods adopted for emotional profiling. The Section 3 articulates a quantitative answer to the research question described in the foregoing section. The Discussion identifies key outcomes and limitations of this study, providing also links to future research using emotional profiling in clinical cognitive science.

## 2. Methods

### 2.1. Corpus of genuine suicide notes

This study adopted the corpus of Genuine Suicide Notes developed and maintained by Schoene and Dethlefs (2016), which includes 143 different suicide notes written by individuals who successfully committed suicide. Notes were written in English, with the names of people changed to random alternatives for privacy purposes. No personally identifiable information was present in the considered textual dataset. Suicide notes consisting of less than two sentences were not considered in the analysis, leading to 139 notes being used in the current analysis. On average, a suicide letter included only 120 words (including stopwords). No contextual information (e.g., socio-economic demographics) was available from the dataset. All the text from different notes was processed together in order to obtain a single cognitive network representing conceptual associations, ideas and emotions as occurring in the minds of people who committed suicide.

The last sentence of each suicide note contains the very last words that a person wrote before successfully ending their lives, a farewell that is worthy of further investigation. In our corpus, these last sentences contained an average of 16 ± 2 words, including stopwords.

### 2.2. Language processing and network construction

This study used two different network construction methods for representing conceptual associations as embedded in suicide notes, namely: (i) word-word co-occurrence networks and (ii) emotional co-occurrence networks.

#### 2.2.1. Word-word co-occurrence networks

Co-occurrences capture relationships between words being close in sentences (Stella, 2022). Word co-occurrences remain computationally lightweight models for parsing how words were used in texts (Amancio, 2015; de Arruda et al., 2019; Ferrer-i Cancho and Gómez-Rodŕıguez, 2021).

Analogously to what was done in a previous approach (Teixeira et al., 2021), we built a network of word-word co-occurrences, where two words were connected if one occurred immediately after the other in at least one sentence of the considered textual corpus. Notice that these networks include a variety of different words, e.g., entities (“family”), verbs (“give”) and even pronouns (“she”) and stopwords (words without meaning when considered in isolation like “of”). In our analysis we kept negations in order to account for them in emotional profiling (see also next section).

For Study 1, we built 2 networks of word-word co-occurrences: one considering all text present in the whole corpus (with 2,075 nodes and 8,676 links) and another considering only the last sentence of every suicide note in the corpus (with 204 nodes and 342 links). Both these networks are fully connected. The co-occurrence network extracted from the whole corpus featured 426 negative words, 1,054 positive words and 595 neutral words. The network of suicide notes featured a Kendall Tau correlation between the sentiment polarities of words connected by links equal to κ_*t*_ = 0.053 (*p* < 0.01), indicating a weak tendency for authors to assemble together concepts that have the same sentiment polarity (e.g., negative words with negative words)—a phenomenon often referred to as *homophily* (Stella et al., 2019).

#### 2.2.2. Emotional co-occurrence networks

In Study 2 we focused on extracting the emotional content of suicide notes, focusing our attention on the co-occurrence of emotional words within the same suicide letter.

A dictionary *D* of emotional words was extracted from a recent experiment using the Emotional Recall Task led by Li and colleagues (Li et al., 2020b). In the experiment, 200 individuals recalled their emotional states in the last month and produced exactly 10 words describing it within a fluency task. The resulting dataset included 475 different stems of emotional words (e.g., happy, sad, etc.) in English. Word stems were considered in order to have only a single representation of lemmas in the network despite word declination (e.g., sad and sadness being both present in the data and both representing the stem “sad").

We then processed the suicide notes. For each letter in the corpus, we: (i) stemmed all words in its sentences, (ii) removed all words outside of the emotional dictionary *D* and (iii) preserved the ordering of emotional words in the letter. Punctuation was discarded. This procedure transformed the 139 suicide notes in the corpus into 139 ordered lists of emotional words. Only 119 lists contained at least 5 emotional words. Five was selected as a threshold matching an analogous number of total responses as observed in the ERT data. We denote the set of these lists as {*L*}. At the overall level of the suicide note, these lists of emotional words contain information about the order and type of emotional ideas that were mentioned by authors while recalling and writing down their final thoughts. In this way, we considered these lists as the outcome of a language production process, certainly more structured than in word recall and fluency task experiments but also tightly connected with the recall of concepts and emotions surrounding the idea of suicide.

Hence, we considered the ordered lists {*L*} of emotional concepts as word sequences coming from suicidal ideation, and represented conceptual relationships among them by applying Goni et al.s' procedure of network construction from word lists (Goñi et al., 2011). The procedure considers co-occurrences of words in each list within a given window and then builds network connections between concepts that co-appeared more often than random expectation. We used a window of size *a* = 2, considering 2 words before and (2 words) after each entry. Links were built by counting all co-occurrences happening at least twice in the dataset and further statistical filtering was applied by considering a Binomial distribution accounting for word frequency in the dataset. For more details, we refer to the original paper (Goñi et al., 2011).

This procedure represented the co-occurrence of emotional jargon across suicide notes as a complex network including 179 nodes/emotional words and 303 links. We investigate the structure of this network and its emotional complexity in Study 2. For comparison, we build a network based on the ERT task through the same methodology outlined above. However, from the ERT data we consider only word lists produced by individuals with no signals of anxiety, stress or depression symptoms. We operationalised this by considering only responses coming from individuals who achieved a total score of 0 on the Depression Anxiety Stress Scales (DASS) (cf. Lovibond and Lovibond, 1995; Li et al., 2020b) in the ERT task. Importantly, we refer to these participants as “mentally healthy.” Notice that the DASS uses a four-point Likert scale, ranging from 0 (never) to 3 (most of the time), indicating how frequently individuals went through specific experiences (items) in a recent time window.

### 2.3. Cognitive datasets and emotional profiling

Words were given both a sentiment label and an emotional attribute. A sentiment label indicates whether a word is in the lower quartile (lower valence/negative perception), in the middle quartiles (neutral valence/perception) or in the upper quartile (higher valence/positive perception) of the distribution of valence scores as obtained from Mohammad (2018). Sentiment labels were used in order to reconstruct the overall sentiment expressed in the considered textual corpus. Sentiment patterns were detected by counting words with positive/negative/neutral sentiment labels.

Emotional profiles were built by considering which emotions were elicited by a given word according to the NRC Emotion Lexicon (Mohammad and Turney, 2013). Individuals enrolled in a mega-study rated which emotions were inspired by individual words. For example, “pandemic" elicited fear. The emotional profile of a text was extracted by considering how many of its words elicited a certain emotion. We call emotional richness *r*_*i*_ the fraction *r*_*i*_ = *m*/*N* of *m* words inspiring emotion *i* in a text composed of *N* words. By definition *r*_*i*_ ranges between 0 (no word in the text elicits emotion *i*, emotional absence) and 1 (all words elicit emotion *i*, complete emotional richness). Notice that a single word can elicit multiple emotions. The collection of observed emotional profiles _*r*_*i*_*i*_ constitutes the so-called *emotional profile* of a text.

The above approach might look like a bag-of-words approach, where a text is considered as the sum of its words (Mohammad and Turney, 2013; Mohammad, 2016). However, this is not true, as in our case the network structure reflects aspects of how words are combined together in text and makes it possible to take into account some mechanisms of meaning modification, such as negations. For both emotional profiling and sentiment identification in the co-occurrence networks, the antonyms of words linked with meaning negations were added to the count of sentiment labels *m* in *r*_*i*_ [e.g., “bad” would be used to represent the phrase “not good,” and would have a negative sentiment label; antonyms were defined *via* WordNet 3.0 (Fellbaum, 2012)].

In order to compare the observed emotional profiles to a baseline, random word sampling was considered. A collection of 1,000 random emotional profiles was built in order to obtain z-scores estimating how likely it was to observe a given emotional profile through random sampling (significance level of 0.05). The collection of 8 z-scores obtained in this way, each one relative to a specific emotion, was then plotted in a sector bar chart visualization resembling a flower layout (see Figure 2). The rejection area *z* < 1.96 was plotted as a semi-transparent circle; each emotion was represented as a petal-shaped bar and concentric circles indicated units of z-scores after 2, providing a visual impression for those emotions more prominently featured in a given text, analogously to the well-known wheel of emotions by Plutchik (1980). We call this visualization an *emotional flower* and it was introduced in Stella (2022).

### 2.4. Cognitive networks and semantic frames

The combination of word co-occurrences, sentiment and emotional profiles constitute three dimensions outlining the content of a given text. These dimensions are built in an automatic way and provide information on the semantic content (network structure), the overall sentiment (pleasantness) and emotional features of the jargon used in text. The combination of knowledge structure and emotional perceptions embedded in texts provides a rich characterization for the content of a given text in terms of quantitative measures (see also Stella, 2022). As an example, the network neighborhood of a given concept *c* identifies words directly co-occurring with *c* in text, thus approximating the so-called semantic frame of *c* itself, i.e., a collection of conceptual units characterizing the meaning attributed to *c* in language. Further characterizing a given semantic frame (Fillmore, 2006) by measuring the sentiment and emotions of its words can reconstruct the emotional perception attributed to *c* in the text itself.

This combination enables a powerful, automatic identification of the semantic frames and emotion perceptions affecting specific stances in a text or in its different sub-components. This automatic structure is a quantitative and approximated reflection of the cognitive component used by the author for writing down that text, i.e., their mental lexicon, the cognitive system appropriate to language acquisition, storage and use (Hills et al., 2015; De Deyne et al., 2016; Vitevitch, 2019; Li et al., 2020b; Mehler et al., 2020; Stella, 2022). In this way, exploring the network of conceptual/emotional patterns extracted from a text is a way of opening a window into people's minds.

### 2.5. Networks can quantify conceptual prominence

Conceptual prominence is measured here through a network approach relying on closeness centrality (Siew et al., 2019), namely:


(1) $$\begin{array}{l} C_{i}=\frac{N-1}{\sum_{j}d_{ij}}, \end{array}$$

where *d*_*ij*_ is the shortest path length between nodes *i* and *j* in a connected component (made of *N* nodes). Closeness indicates the inverse average distance between one node and all of its connected neighbors. Nodes connected to their neighbors by few (many) links, on average, will possess higher (lower) closeness centrality. In networks of word-word relationships, Stella (2022) showed that closeness centrality was powerful in identifying words expressing the topic of short texts. In networks of free associations, recent work by Kenett et al. (2017) and replicated by Kumar et al. (2020) showed that network distance, which closeness is based on, was more powerful than latent semantic analysis in predicting semantic similarity rates in empirical experiments. Lastly, closeness centrality was highly predictive of words prominently featured in early learning environments and learned by young children during early word acquisition (Stella et al., 2017).

### 2.6. Capturing the emotional complexity of sequences of words

Keeping in mind that in an emotional network all nodes represent jargon relative to one (or more) emotional sphere(s), then links can be interpreted as transitions between emotional states of different valence. Emotional networks like the one introduced here can be applied to the quantification of *emotional complexity* in texts through two synergistic approaches. With the aim of understanding how these emotional aspects of language were expressed in language by those who committed suicide and authored suicide notes, we introduce two concepts:

- **Emotional entropy**, i.e., the Shannon entropy of transitions between positive, negative and neutral emotional states as encountered in suicide notes and as detected by the emotional recall task (Li et al., 2020a). This metric is network independent.
- **Emotional complexity**, i.e., a translation of structural balance theory (Moradimanesh et al., 2021) into the domain of cognitive networks, where links are signed not according to social ties, but rather according to the valence of words connecting them. Thus, signed links indicate connections or flows of emotional jargon in the overall narrative of suicide notes. This metric is network dependent.

The emotional network built here encapsulates information about the flow of emotions in the narratives provided by individuals who committed suicide. Emotionally complex stories rarely describe emotional states of the same valence, as they may transition frequently between positive and negative emotional states such as sadness and hope. Stories with lower emotional complexity, however, will tend to switch less frequently back and forth between positive and negative emotional spheres. This difference can be captured by the emotional entropy, i.e.,:


(2) $$\begin{array}{l} h_{n}=-\underset{i}{\sum }p_{i}\log\left(p_{i}\right), \end{array}$$

where *p*_*i*_ is the probability of occurrence of event *i*. We sum over two mutually exclusive events, namely that two words appearing one after another in a list of emotional words *n*∈{*L*} have equal valence (event 1) or not (event 2). Methodologically, *h*_*n*_ depends on counting how many transitions between valence labels (positive, negative or neutral) occur between adjacent words in an ordered list *n*∈{*L*}. Let us consider an example, e.g., lists *n*_1_ = {sad,sick,fine,great,normal}. The valence data used here and based on the Emotional Lexicon (Mohammad, 2018) would map *n*_1_ into the sequence of emotional labels {negative,negative,positive,positive,neutral}. Starting from the first position and placing a 0 when the subsequent label is the same and 1 otherwise, *n*_1_ would then be mapped into the binary string {0,1,0,1}, so that *h*_*n*_1__ = −1/2log(1/2)−1/2log(1/2) = log2. Using a binary logarithm, then *h*_*n*_1__ = 1. Notice that this definition depends on how many emotional transitions appeared in a given list. Let us consider a second example, *n*_2_ = {great,sad,fine,sick,normal}. This list would be mapped in {positive,negative,positive,negative,neutral} and consequently in {1,1,1,1}. Despite *n*_1_ and *n*_2_ having the same set of words, the entropy of *n*_2_ would be *h*_*n*_2__ = 0log0+1log1 = 0, far lower than *h*_*n*_2__. Because of its functional form, *h*_*n*_ captures whether emotional transitions are frequently present in a list and scattered uniformly across it (higher entropy) or rather occurring together (lower entropy).

Each suicide note transformed into a list *n* corresponds to an entropy *h*_*n*_. Analogously, each word list *m* in the ERT dataset gives rise to an entropy *h*_*m*_. Statistical testing between the samples of entropies from the suicide notes _*h*_*n*_*n*_ and from the recall task _*h*_*m*_*m*_ enable further comparison between notes and recall lists.

In addition to comparing suicide notes and recall lists in terms of the entropy with which positive/negative/neutral emotional states appear in them, we also performed comparisons between each corpus and a randomized version of it. We designed two null models:

- A Shuffled model, preserving the length of each list, the dictionary of words present in these lists and their valence, but reshuffling the order in which words appear in the list;
- A Uniform model, preserving the length of each list and the dictionary of words present in these lists, but reshuffling the order in which words appear in the list and replacing their empirical positive, negative or neutral valence with a fictional value selected uniformly at random. This model disrupts correlations between valence and other quantities (e.g., frequency of positive words in a list).

A higher entropy, i.e., higher than random expectation, will be indicative of more uniformly random and less coherent transition rates between positive, negative and neutral words in a given corpus (suicide notes or ERT data).

#### 2.6.1. Focus on emotional complexity as a network measure

We draw inspiration from psychology to operationalize emotional complexity as a network metric that quantifies how positive and negative emotional states tend to be associated or contrasted with each other in text. Building on important past attempts like Van Rensbergen et al. (2015)'s emotional assortativity, Stella et al. (2019) emotional homophily, and Teixeira et al. (2021) emotional balance, which all quantified the tendency for free associations to link words sharing the same positive, negative, and neutral valence in language, emotional complexity considers how triangles of conceptual associations tend to mix emotional words with opposing emotional polarities, e.g., negative and positive emotional states.

A network of linked emotional states will be considered as structurally incoherent, i.e., with a higher emotional complexity, whenever the text underlying that network contains frequent switches between positive and negative emotional jargon along the flow of narration.

**Figure 1:**
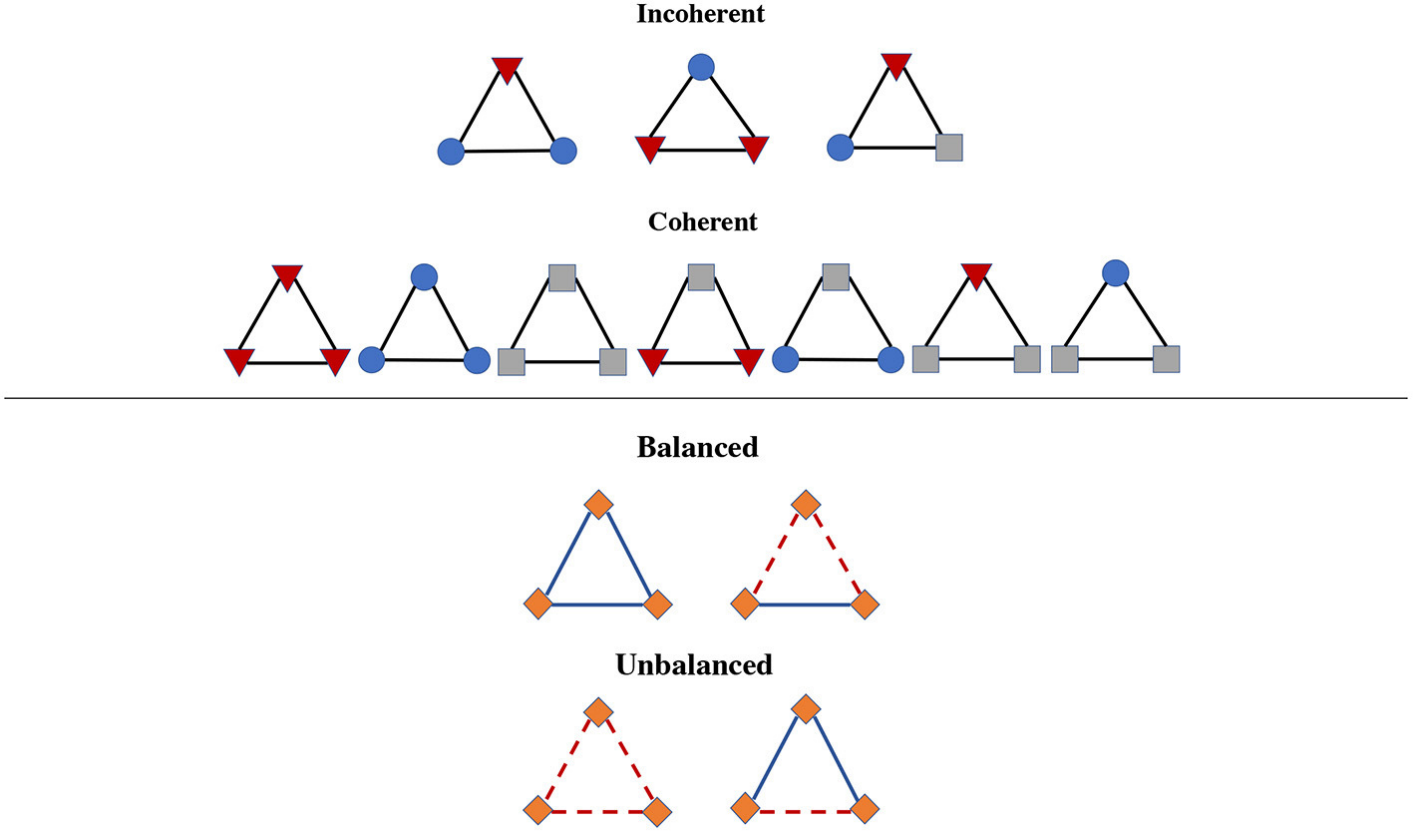
Comparison of coherent/incoherent triads for measuring emotional complexity in word networks **(Top)** vs. balanced/unbalanced triads for measuring structural balance in social networks **(Bottom)**. Shapes indicate positive (circles), negative (triangles), or neutral (squares) words. Whereas, emotional coherence is based on nodes and words (incoherent triads do not mix words of opposite valence), structural balanced is based on positive and negative links, instead.

Figure 1 compares emotional complexity and structural balance theory applied to networks featuring positive, negative and neutral nodes (e.g., enemies, friends or neutral agents or positive, negative and neutral words in language). Differently from structural balance theory, in our case it is the properties of the *nodes*, rather than the *edges*, that are used to distinguish (in)coherent triads: Incoherent triads contain at least one word with negative valence and one word with positive valence, while all other triads are coherent.

Analogous to the degree of balance in structural balance theory—i.e., the fraction of balanced triangles in a given network configuration—we here introduce the degree of emotional complexity *c* for a network as the fraction of emotionally incoherent triads:

(3) $$\begin{array}{l} c=\frac{n_{i}}{n_{c}+n_{i}}, \end{array}$$

which is the complement to 1 of the fraction of coherent triads. Notice that “complexity" for us should then be interpreted as a measure of the *lack* of emotional coherence. For this reason, in the visualizations for this work we will focus on coherence, 1−*c*, while for the more appropriate psychological interpretation of the measure we will consider complexity, i.e., *c*. This difference is motivated by the fact that the term “emotional coherence" already appears in other psychometric approaches. Notice that in our definition we consider neutral words as not giving rise to any contrast with either negative or positive words.

Please notice that emotional complexity and entropy, as described above, can be interpreted through comparisons between two or more letters. At the network level, these measures are devised to compare quantitatively how the overall narrative of suicide notes structures emotions compared to clinically healthy emotional recollections. Distributions of emotional entropies/complexity scores might differ in their medians between healthy adults and authors of suicide notes but variability in each distribution would make it difficult to interpret patterns for individual recalls/notes. To achieve individual-level granularity, we would need considerably more data, particularly in terms of several independent measurements or texts produced by individuals who ended their lives. Given the sensitive nature of this data, e.g., a genuine suicide note is the last text produced by a person before they end their life, this limitation poses challenges that go beyond this study. For this reason, we will keep our analyses and discussions at group-level.

Notice also that counting triads with emotional complexity provides different information compared to the entropy estimates. The latter capture rates of transitions between positive, negative and neutral emotional words, as discussed in Section 2.6. However, transitions are pairwise and do not account for the tendency for groups of writers/recallers to go back to positive/negative/neutral after having transitioned to any other pairwise combination. This difference makes triads, as counted in emotional complexity, more insightful about the clustering of emotional words in the set of suicide notes.

#### 2.6.2. Null models for emotional complexity

Analogous to our approach with emotional entropy, in order to identify whether the abundance of coherent/incoherent triangles in the emotional network is statistically significant we identify two different null models which preserve the empirical network structure (i.e., the number of triangles observed in the real network) but reshuffle the valence of individual words in triangles. Additionally, these null models are designed to:

- preserve the frequency of positive/negative/neutral words in the empirical emotional network (Shuffled model);
- make positive/negative/neutral words occur uniformly at random (Uniform model).

Across 500 iterations of each null model, we measured z-scores for all empirical triangle counts and fixed a significance level of 0.05 in order to identify those triangles over- or underrepresented in the network (*z* < −1.96 or *z* > 1.96). For each iteration *r* we also measured the degree of coherence *c*_*r*_, thus enabling a statistical comparison between the empirical value observed in the emotional network *c* and a distribution of coherence values for each null model.

## 3. Results

Results are split in two studies. Study 1 focuses on understanding how the final sentences of suicide notes are structured and which emotions populate them in comparison to the other parts of such texts. Study 2 focuses on highlighting the emotional words of suicide notes in view of suicidal ideation and by using data coming from the emotional recall task for the first time.

In the following we refer to all words included in the last sentence of each suicide note as the “last words", outlining the closing remarks of each text.

### 3.1. Study 1: Prominence and emotional content of last words in suicide notes

This section aims to quantify how prominent or peripheral the last words were within the overall landscape of conceptual associations expressed in the suicide notes. The last sentence of each genuine suicide note represents a final farewell produced by the author. These sentences included on average 16 ± 2 words, including stopwords.

**Figure 2:**
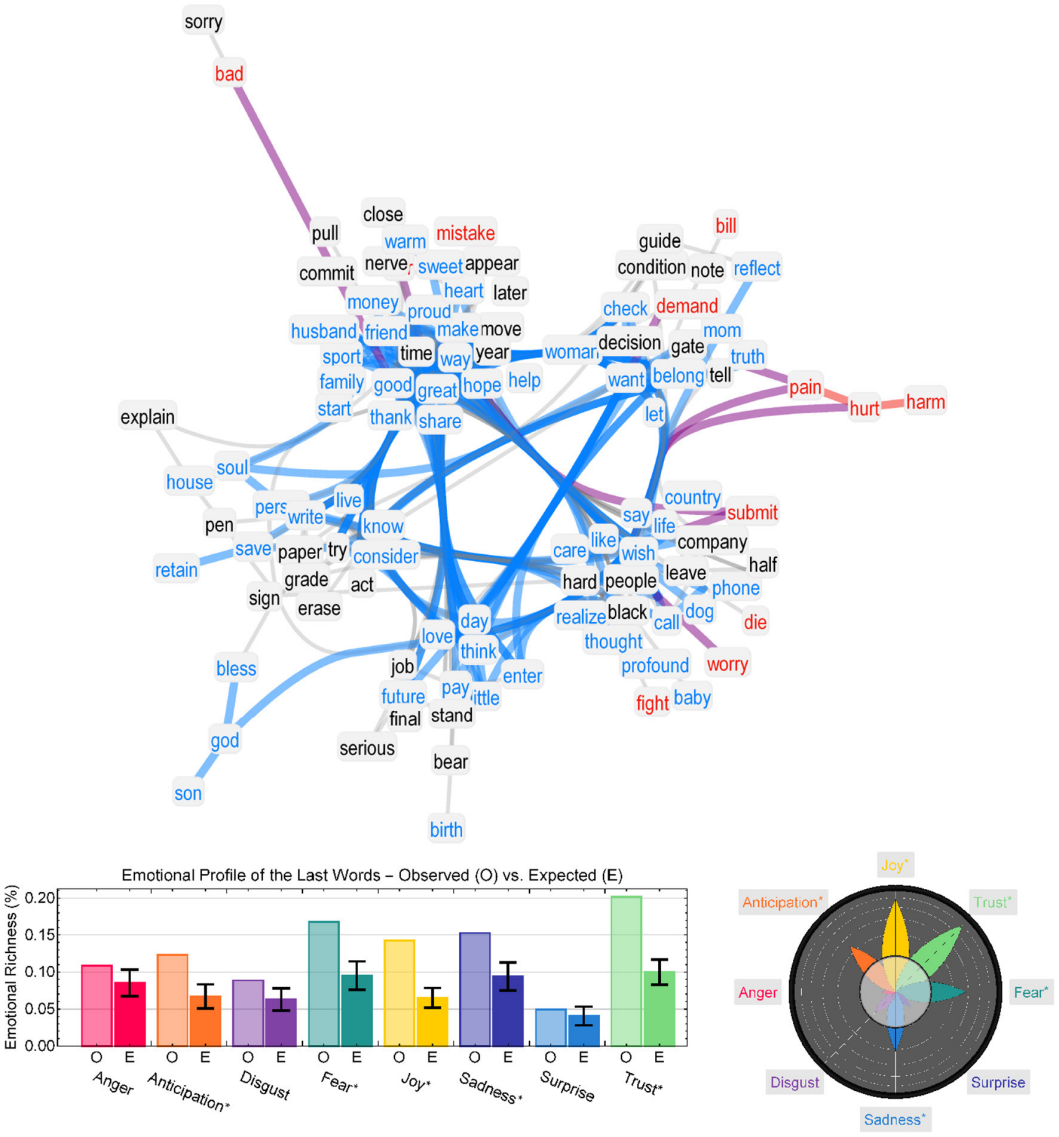
Network visualization of the largest connected component of co-occurrences between last words as expressed in the suicide notes. Cyan highlights positively perceived words whereas red highlights negative concepts. Links between positive (negative) concepts are in cyan (red). Co-occurrences between positive and negative (neutral) concepts are in purple (gray). Co-occurrences between neutral words are thinner for visualization purposes. Neutral words are in black. **(Left)** Emotional profiles of last words observed in suicide notes against the expected profile of 1,000 random samples of 204 non-last words. **(Right)** Emotional flower reporting positive z-scores between the observed emotional profiles of last words and random expectation of non-last words.

The considered corpus included a total of 204 last words, excluding stop-words and names of people or places. This means that on average each last sentence brought to the network roughly 204/139 ≈ 1.5 concepts/words. This statistic suggests a tendency for the final farewells in suicide notes to revolve around coherent topics, which we can explore through network analysis and emotional profiling. Seventy-five of the unique words in the network extracted from last sentence were positive while 39 of them were negative. Figure 2 presents a network visualization of the largest connected component of the network of last words as co-occurring with each other in the suicide notes. For easier visualization, words were clustered according to the Louvain algorithm (Blondel et al., 2008), based only on text co-occurrences. The network visualization highlights five clusters of words used by authors in the closing remarks of their suicide notes. One cluster features spiritual concepts like “soul,” “bless,” and “god” among other non-spiritual terms like “pen,” “paper" and “erase.” Another cluster associates several positive concepts to “time" and “family.” Although dominated by concepts generally perceived as positive, the last words also included negative concepts like “harm,” “pain,” and “hurt.” These patterns indicate a tendency for the ending remarks to express concepts of emotional positivity but also emotional pain. But, are these concepts peripheral or prominent in the whole text portrayed by authors of suicide notes?

To address this question we measured the closeness centrality of each final word in the complete network of word co-occurrences as extracted from all the considered notes. These 204 centrality scores were then compared against the closeness centrality of all 1,871 other words in the network. A statistical test identified the last words as being on average more central, i.e., with a higher closeness centrality, than all other words in the suicide notes (Kruskal-Wallis, *N*_1_ = 204, *N*_2_ = 1871, 77.98, *p* < 0.001). This difference indicates that the words used by the authors for closing their notes are most prominent in the whole discourse revolving around suicide.

No statistically significant difference was found between last words and non-last words in terms of sentiment polarity, i.e., final and non-last words were similarly distributed among positive/negative/neutral categories (χ^2^ = 5.54, *p* = 0.622 > 0.05).

The finer categorization of words across emotions highlighted statistically significant differences. The emotional profile of all 204 last words was compared against 1,000 random samples of 204 non-last words drawn from the largest connected component of the co-occurrence network built on all sentences in suicide notes (Teixeira et al., 2021) excluding stopwords. In this way, we compared the emotional content of conceptual associations present in the last sentences of suicide notes against those links present in other parts of suicide notes. By sampling words from a network of co-occurrences rather than directly from the text, we take into account connectivity between concepts in the absence of stopwords. The resulting z-scores, comparing the emotional profiles of final and non-last words, are reported in Figure 2. As reported in Figure 2, bottom, last words elicited anger, surprise and disgust to the same extent as other words in suicide notes. In comparison to other concepts recurring in suicide notes, the 204 unique last words, identified here, elicited stronger patterns of sadness and fear, but also joy, trust and anticipation.

### 3.2. Study 2: Emotional content and emotional complexity of emotional words in suicide notes

This study focuses on using two measures of emotional coherence (see Methods) for the investigation of emotional switches between positive, negative and neutral emotional states in suicide notes.

#### 3.2.1. Emotional entropy

Healthy individuals and suicide letters exhibited analogous entropy levels (Kruskal-Wallis, *K* = 1.958, *p*≥0.162), indicating no differences in emotional transitions between recalls by healthy individuals and narratives in suicide letters.

A comparison of the emotional entropy of suicide notes against the reference nulls models (see Methods) identified the key role of word frequency in modulating the switches observed in suicide notes between positive, negative and neutral words. Both null models disrupted emotional associations by randomly reshuffling the same emotional states present in suicide notes. However, one null model preserved the empirical word frequency of emotional states while another implemented emotional states all with the same occurrence rate.

Considering 500 random iterations, the median entropy observed in suicide notes was lower than random expectation only for the null model discarding concept frequency (Kruskal-Wallis, *K* = 11.082, *p* ≤ 0.0004) but not for the null model preserving word frequency (Kruskal-Wallis, *K* = 1.261, *p*≥0.262). This indicates that the empirical frequency of emotional states and the empirical length of suicide notes (in terms of emotional words) are sufficient to produce the same rate of switches observed in the empirical suicide notes. Let us consider this more deeply. We observe a certain sample or lexicon of words, i.e., a collection of words, each one either neutral or negative or positive. How much of the entropy observed in word lists could be explained by the affective composition of the lexicon? For instance, a lexicon fully composed of positive words would give rise to word lists fully made of positive words and thus with 0 entropy. In case of a lexicon combining all sentiment polarities, we would expect a higher entropy. Our testing tells us that such expected entropy, obtained by sampling words in lists uniformly at random from the lexicon, is still different from the entropy we observe in the empirical word lists. Hence, the composition of the lexicon in terms of positive/negative/neutral words is not sufficient to generate the emotional transitions we observe in the empirical data. Instead, sampling words at random but according to their frequency provides entropic rates of switches between emotional states that are compatible with the entropies observed in the empirical data. In other words, counting adjacent emotional transitions through entropy highlights no evident difference between frequency-based random models and suicide notes. The frequency with which emotional words were used in suicide letters is crucially enough to reproduce emotional transitions analogous to the empirically observed ones. Going beyond counting adjacent transitions might highlight potential differences.

We turn to a network analysis of emotional complexity tracking how positive and negative emotional states are associated in suicide notes.

#### 3.2.2. Emotional complexity

Considering only emotional suicide notes with at least 5 emotional words leads to similar numbers of triads for suicide nodes (554) and healthy recalls (528), with a ratio of 554/528 or 105%.

A preliminary measure of emotional complexity can be counting balanced and imbalanced triads individually present in each suicide letter/recall. Healthy recalls (i.e., by individuals with no level of stress, anxiety or depression) featured 127% as many emotionally coherent triads (see Figure 1 for the definitions of coherent and incoherent triads) as suicide letters, i.e., 362 vs. 284. Instead, suicide letters featured 163% as many incoherent triads as healthy recalls, i.e., 270 vs. 166. In particular, suicide letters featured almost 346% as many incoherent +, −, + triads (with fixed order) as healthy recalls, i.e., 45 vs. 13. Similarly, suicide letters featured almost 171% as many incoherent −, +, − triads (with fixed order) as healthy recalls, i.e., 24 vs. 13. These counts indicate that, when considered individually, suicide notes featured more emotionally incoherent sequences of negative/positive words compared to health recalls. This is a first indication that by considering triads rather than adjacent transitions, structural differences in the emotional structure of suicide narratives can arise. After checking that individual suicide narratives can give rise to more emotionally incoherent patterns than healthy recalls, we use a network framework to test whether these patterns persist when emotional transitions are aggregated together and perform more statistical comparisons against random null models.

The network of emotional transitions extracted from suicide notes featured mostly incoherent +, +, − (occurring 80/278 times) and coherent +, +, + triangles (occurring 77/278 times). In order to assess whether the observed occurrences are peculiar, comparisons with suitable null models are required (see Methods). We compare emotional coherence in suicide notes and in the ERT data from mentally healthy individuals (i.e., individuals achieving a score of 0 in the DASS psychometric scale or, equivalently, no self-reported sign for anxiety, depression and stress) by considering two distinct networks (see Methods). For each network we also build random null models by either reshuffling word valence while preserving valence frequency and network structure (Shuffled null models), or by giving words fictional valence labels (positive/negative/neutral) uniformly at random (Uniform null models).

Notice that the set of emotional words extracted from suicide notes was a subset of the emotional states identified by the ERT data. In suicide notes, negative emotional states occurred 31% of the time, positive states were mentioned 52% of the time while the remaining 17% was composed of neutral emotional states. In the ERT data provided by mentally healthy individuals, negative emotional states occurred only 22% of the time, positive states were mentioned 48% of the time, and neutral words occurred 30% of the time. These differences might give rise to different patterns of emotional complexity that should be further investigated.

**Figure 3:**
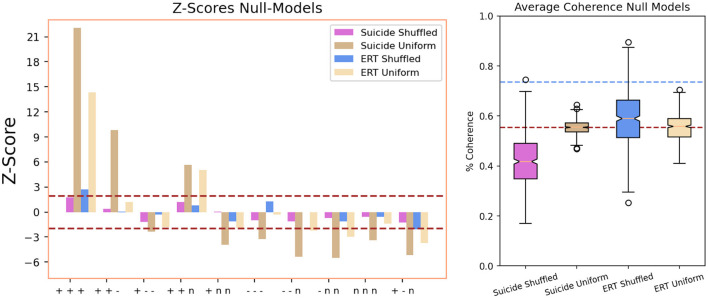
**(Left)** Z-scores indicating the occurrence of signed triangles in the empirical emotional networks for suicide notes and ERT recalls against 2 random null models (Shuffled and Uniform). **(Right)** Boxplot comparing the distribution of observed emotional coherence in the null models against the value observed in the emotional network of suicide notes (dashed lines, the lower one is relative to suicide notes, the upper one to healthy ERT recalls).

Figure 3, left shows that the occurrence of coherent +, +, + triangles in the emotional network of suicide notes is compatible with random expectation when reshuffling word valences while preserving valence frequency (Suicide Shuffled model, *z* < 1.96). In comparison to reshuffling word valences with uniform valence frequencies, suicide notes feature more +, +, + triangles than expected (Suicide Uniform model, *z* > 1.96). Analogous patterns are observed for the +, +, − and +, +, *n* triangles. These differences indicate that the frequency with which emotional states are mentioned in suicide notes is sufficient for determining a high count of +, +, +, +, +, − and +, +, *n* triangles, suggesting there is no evident influence of network structure in suicide notes on the formation of closed triads of positive emotional states. This is not the case in the ERT network, however, where a higher occurrence of +, +, + triangles is observed in comparison with both the Shuffled (*z* = 2.9 > 1.96) and Uniform (*z* = 15.2 > 1.96) null models. This difference indicates that the observed count of fully positive triangles in the emotional recall from mentally healthy individuals (i.e., individuals achieving a score of 0 in the DASS psychometric scale or, equivalently, no self-reported sign for anxiety, depression and stress) cannot be fully accounted for by considering only valence frequency, but instead requires both valence frequency and network structure to obtain a complete picture.

For other triangles we do not observe differences between the empirical networks and the Shuffled null models. However, notice that these analyses assume that each individual triangle count is a random variable independent of others. In the Shuffled null model, since the empirical frequency of positive/negative/neutral concepts is preserved, triangle counts cannot evidently be treated as independent and this calls for additional analysis in terms of coherence assessment.

Hence, we shift our attention from individual triangle counts to the measurements of emotional complexity *c* (see Section 2). Figure 3, right reports the value of *c* for the emotional suicide network (brown dashed line, *c*_*S*_ = 0.55) and for the healthy ERT network (blue dashed line, *c*_*S*_ = 0.71). Healthy individuals devoid of stress, anxiety and depression (based on their DASS subscale scores) produced conceptual associations between mental states with a coherence almost 30% higher than the one observed in emotional associations from suicide notes. Combining this result with the finding that healthy individuals recalled neutral emotional states almost twice as frequently as suicide notes, as well as considering how neutral triangles contribute to our definition of emotional complexity, the observed difference indicates that healthy individuals tend to feature more elementary cycles—i.e., triangles—of neutral emotional states in comparison to the authors of suicide notes.

Our null models can test two potential sources of emotional complexity: (i) valence-network correlations between words, e.g., trends for words of similar valence to originate cycles together, (ii) valence frequency, e.g., positive words being more frequent than negative ones and creating more coherent triangles.

Disrupting any correlation between the valence of a concept and its network location reduces the overall emotional coherence in both the considered networks. Even when preserving the empirical frequencies of valence, the Shuffled models produce networks with evidently lower median emotional complexity than their respective empirical values. Hence, valence frequencies by themselves are not enough to reproduce the emotional coherence observed in both the suicide and ERT emotional networks. These patterns of emotional coherence are therefore to be attributed to an interplay between affect and network structure which ultimately makes emotional shifts in either suicide notes or recall tasks less “switchy" and more confined within elementary cycles of words sharing the same valence.

Disrupting the empirical valence frequency in the observed network leads to the same average value of emotional complexity. Notice how preserving valence frequency also preserves the observed gap in emotional complexity between suicide notes and healthy individuals. This indicates that whereas the coherence of each network stems from correlations between network structure and valence, the observed gap in coherence between suicide notes and the ERT data has to be attributed mainly to a different frequency of positive/negative/neutral emotional states.

To summarize, the above results indicate that the narratives in suicide notes are more structurally coherent than expected by a null model where valenced words are sampled according to the same frequency observed in the empirical dataset but independently from network structure. Furthermore, the narratives in suicide notes are less emotionally coherent than observed in emotional associations provided by healthy individuals, a difference that can be explained by a higher occurrence of neutral emotional states in healthy individuals in comparison to suicide notes.

## 4. Discussion and conclusions

This study reconstructed the associative knowledge expressed by authors of suicide notes (Schoene and Dethlefs, 2016; Boyd, 2017) using graph theory and network science (Newman, 2018). This mindset was represented as a network of word co-occurrences in texts enriched with emotional data. The network structure enabled a quantitative comparison between different groups of words. The last words used by authors at the end of their letters were shown to be highly prominent concepts in the overall discourse of suicide notes, as quantified by closeness centrality. Authors might therefore use these last words in order to reinforce their narrative, repeat, and summarize their points or express general-level concepts highly relevant to what they described in their notes. In line with recent advancements in natural language processing using cognitive data for improving model interpretability (Škrlj et al., 2021), the cognitive network structure provides a proxy of the associative knowledge between such words in text. Semantic analysis reveals that these prominent last words are mostly clustered around the topics of family, religion and painful emotional processing, in agreement with previous human coding studies of suicidal ideation (Sanger and Veach, 2008). In comparison to other less prominent concepts expressed in suicide notes, last words elicited more intense feelings of sadness and fear, but also elicited joy, trust and anticipation.

Trust and joy are emotions in line with the above semantic analysis exposing clusters of family-related jargon in the closing remarks of suicide notes. The emotional profiling reported here indicates that the closing remarks contain concepts strongly expressing positive emotions, providing a trustful ending to notes. However, in the considered collection of suicide notes, this positive remark co-existed with anticipation, fear and surprise, a combination that expresses uncertainty about the future (Plutchik, 1980). The co-existence of fear and trust in last words can also be related to the above cluster of words which express painful emotional processing. Once combined, these patterns indicate that suicide notes also express jargon of trustful relief from fearful and painful concepts, providing quantitative support to psychological theories indicating suicidal ideation as a pain relief mechanism (Sanger and Veach, 2008; Schoene and Dethlefs, 2016).

This preliminary study outlines the quantitative potential for network science (Newman, 2018) to give insight into the structure of word co-occurrences and their emotional associations (Siew et al., 2019). Network approaches to modeling cognition rely on the assumption that every author possesses a cognitive system, a mental lexicon (De Deyne et al., 2016; Siew et al., 2019; Stella et al., 2019) where concepts have an associative semantic and emotional structure. The approach outlined here might potentially be integrated with natural language processing at the stage of network creation in order to achieve more realistic knowledge representations, expressing syntactic dependencies (Stella, 2022) or sociolinguistic features of texts (Boyd, 2017). Recent work has successfully used co-occurrence networks for the investigation of authorial writing style (Amancio, 2015; de Arruda et al., 2019) and social media discourse (Stella et al., 2018). Co-occurrences were merged with emotional features in recent investigations of social media discourse around the gender gap in science (Stella, 2022) and the COVID-19 pandemic (Li et al., 2020a; Stella et al., 2020), opening the way to future mindset reconstructions of different authors through additional research.

This work also introduced the network metric of emotional complexity in terms of counting triads connecting positive, negative and neutral emotional words. Emotional complexity considers different kinds of signed triangles in comparison to structural balance. In structural balance theory, triangles are identified as unbalanced whenever there is an odd number of negative links, i.e., an odd number of tense relationships. This rule captures tension in social connections and is a phenomenon supported by relevant social science literature (e.g., Doreian and Mrvar, 2015; He et al., 2018; Aref and Wilson, 2019). One possibility is to extend structural balance also to valenced words, as was done in previous work (Teixeira et al., 2021), in order to quantify tension in the outline of conceptual associations featuring negative words. However, when considering words made of emotional states only, as in the present case, it is possible to shift the attention from an overall level of tension between concepts to the specific tendency for elemental cycles in the network to feature transitions between emotional states of opposing valence. This shift is the key difference between the current approach with emotional complexity and the one by Teixeira and colleagues in terms of emotional balance (Teixeira et al., 2021). Whereas, in emotional balance triads are categorized according to their links, in emotional complexity triads are classified according to the valence of their nodes. Configurations that are emotionally balanced can end up being treated as incoherent in terms of emotional complexity. Considering coherence/balance in terms of nodes rather than in terms of links is evidently different from structural balance and more akin to emotional complexity in cognitive psychology (Bagozzi et al., 1999; Scollon et al., 2005; Barrett, 2006). In other words, whereas structural balance focuses on the positive/negative nature of social ties—operationalising the balance of concepts like “the enemy of my enemy is my friend"—emotional complexity focuses on the valence of individual emotional states. As opposed to social ties, when considering emotional complexity and its network counterpart a triangle connecting two negative and one positive emotional concept must be considered as incoherent, as it captures an emotional transition between negative and positive emotional states.

The emotional complexity (coherence) of suicide narratives was found to be higher (lower) than that of healthy individuals recalling recent emotions over the past month. Emotional complexity captures a tendency to experience a mixture of positive and negative emotions over time (Diener and Emmons, 1985), and therefore unsurprisingly relates to the time frame within which people recall their emotional experience. That is, people are more likely to report a greater mixture of positive and negative emotions when describing their experience over a long period of time than their experience at the moment (Diener and Emmons, 1985). The higher degree of complexity in suicide notes may suggest that these notes refer to emotions over a long period in life instead of emotions at the moment of writing. Indeed, as we read though the suicide notes, many notes refer both to relatively recent sadness (e.g., “I just can't carry on the despair") and an overview of life in general (e.g., in the same note: “I have looked at our 25 years mainly as happy years and had hope to spend old age together"). Moreover, high complexity in suicide notes may also suggest that people who chose suicide were not simply dominated by negative feelings; instead, they still have access to their positive memories.

These results suggest new topics for further research, particularly to improve an understanding of the relationships between emotional complexity, suicidal ideation (SI), and suicidal behavior (SB). If emotional complexity is predictive of SI or SB, it would be important to study the degree to which it reflects an individual trait or is relatively stable over time. Some clinical psychological studies with a similar aim have shown that affective variability (i.e., the extent to which a person experiences fluctuations in mood) and variability in SI may reflect stable traits (Oquendo et al., 2020) that are associated over time (Rizk et al., 2019), and that affective variability may be an effective predictor of SI (Palmier-Claus et al., 2012). These studies used experience sampling methods to see how such constructs relate over time on a day-to-day scale, however such relationships have only been demonstrated in certain high-risk populations. Extending this work to investigate how emotional complexity and emotional entropy relate with risk of SI or SB would thus be a potentially valuable topic to study in larger populations.

The approach outlined here has several limitations. The most prominent is the necessity of using sentiment and emotional labels extracted from large populations for reconstructing an author's mindset. This methodology neglects subjective interpretations, e.g., high school students might perceive “physics" as a negative concept, whereas large populations might perceive it as neutral (Stella et al., 2019). A way to overcome this limit would be the adoption of language processing techniques identifying the context around a given concept and inferring its emotional perception (Van Rensbergen et al., 2016). This is possible even with cognitive networks by considering valence and emotions in the semantic frame of associates around a concept. In this way, focus should be given not to the specific sentiment or emotional labels attributed to a given word but rather to its structural position among its adjacent neighbors in the text. Another limitation of our study is relative to emotional complexity focusing only on elementary cycles, i.e., closed triads, and disregarding other network patterns that might arise from emotional disassortativity. In order to address this point we considered not only emotional complexity but also emotional entropy, the latter being a non-network metric considering shifts between emotional states in the whole datasets.

Another important caveat of our approach is its generalisability. Suicide letters may be influenced by socio-demographic and cultural elements. This suggests the need for future work that evaluates the role of socio-demographic and cultural elements alongside a larger and more diverse set of suicide notes. Such replication, which is fundamental in psychological research, is facilitated by the current statistical framework presented which can easily be applied to text. Future research could integrate emotional complexity and entropy with recent measures like conformity (Rossetti et al., 2021), mixing network structure and node features for future investigations of larger corpora of suicide notes or other types of texts.

## Data availability statement

Publicly available datasets were analyzed in this study. This data can be found at: https://osf.io/qkyuw/.

## Ethics statement

The studies involving human participants were reviewed and approved by Ethics Committee at Center for Adaptive Rationality, Max Planck Institute for Human Development. The patients/participants provided their written informed consent to participate in this study.

## Author contributions

MS, TS, YL, and AT: data analysis. MS: supervision. All authors: investigation, study design, and writing.

## References

[B1] Amancio D. R. (2015). A complex network approach to stylometry. PLoS ONE 10, e0136076. 10.1371/journal.pone.0136076 26313921 PMC4552030

[B2] Aref S. Wilson M. C. (2019). Balance and frustration in signed networks. J. Complex Netw. 7, 163–189. 10.1093/comnet/cny015

[B3] Baddeley J. L. Daniel G. R. Pennebaker J. W. (2011). How henry hellyer's use of language foretold his suicide. Crisis 32, 288. 10.1027/0227-5910/a000092 21940249

[B4] Bagozzi R. P. Wong N. Yi Y. (1999). The role of culture and gender in the relationship between positive and negative affect. Cogn. Emot. 13, 641–672. 10.1080/026999399379023

[B5] Barrett L. F. (1997). Computing global structural balance in large-scale signed social networks. Pers. Soc. Psychol. Bull. 23, 1100–1110. 10.1177/0146167297231001022167802

[B6] Barrett L. F. (2006). Solving the emotion paradox: categorization and the experience of emotion. Pers. Social Psychol. Rev. 10, 20–46. 10.1207/s15327957pspr1001_2 16430327

[B7] Bayram U. Lee W. Santel D. Minai A. Clark P. Glauser T.. (2022). Toward suicidal ideation detection with lexical network features and machine learning. Northeast J. Complex Syst. 4, 2. 10.22191/nejcs/vol4/iss1/2

[B8] Blondel V. D. Guillaume J.-L. Lambiotte R. Lefebvre E. (2008). Fast unfolding of communities in large networks. J. Stat. Mech. 2008, P10008. 10.1088/1742-5468/2008/10/P1000821517554

[B9] Borsboom D. (2017). A network theory of mental disorders. World Psychiatry 16, 5–13. 10.1002/wps.20375 28127906 PMC5269502

[B10] Boyd R. L. (2017). “Psychological text analysis in the digital humanities,” in Data Analytics in Digital Humanities (Berlin: Springer), 161–189.

[B11] Corrêa Jr E. A. Marinho V. Q. Amancio D. R. (2019). Semantic flow in language networks. arXiv preprint arXiv:1905.07595. 10.1016/j.physa.2020.124895

[B12] de Arruda H. F. Marinho V. Q. Costa L. d. F. Amancio D. R. (2019). Paragraph-based representation of texts: a complex networks approach. Inf. Process. Manag. 56, 479–494. 10.1016/j.ipm.2018.12.008

[B13] De Deyne S. Kenett Y. N. Anaki D. Faust M. Navarro D. J. (2016). “Large-scale network representations of semantics in the mental lexicon,” in Big Data in Cognitive Science, ed M. N. Jones (New York, NY: Routledge; Taylor & Francis Group), 174–202.

[B14] Demirkol M. E. Tamam L. Namli Z. Karaytu,g M. O. Yeşiloglu C. (2022). The relationship among anxiety sensitivity, psychache, and suicidality in patients with generalized anxiety disorder. J. Nerv. Ment. Dis. 6, e295. 10.1097/NMD.0000000000001534 35605224

[B15] Diener E. Emmons R. A. (1985). The independence of positive and negative affect. J. Pers. Soc. Psychol. 47, 1105–1117. 10.1037/0022-3514.47.5.1105 6520704

[B16] Doreian P. Mrvar A. (2015). Structural balance and signed international relations. J. Soc. Struct. 16, 1. 10.21307/joss-2019-01222167802

[B17] Fellbaum C. (2012). “Wordnet,” in Theory and Applications of Ontology: Computer Applications. (Dordrecht: Springer), p. 231–243.

[B18] Ferrer-i Cancho R. Gómez-Rodríguez C. (2021). Anti dependency distance minimization in short sequences. A graph theoretic approach. J. Quant. Linguist. 28, 50–76. 10.1080/09296174.2019.1645547

[B19] Fillmore C. J. (2006). Frame semantics. Cogn. Linguist. 34, 373–400. 10.1515/9783110199901.373

[B20] Goñi J. Arrondo G. Sepulcre J. Martincorena I. De Mendizábal N. V. Corominas-Murtra B.. (2011). The semantic organization of the animal category: evidence from semantic verbal fluency and network theory. Cogn. Process. 12, 183–196. 10.1007/s10339-010-0372-x 20938799

[B21] Hallensleben N. Glaesmer H. Forkmann T. Rath D. Strauss M. Kersting A.. (2019). Predicting suicidal ideation by interpersonal variables, hopelessness and depression in real-time. An ecological momentary assessment study in psychiatric inpatients with depression. Eur. Psychiatry 56, 43–50. 10.1016/j.eurpsy.2018.11.003 30530103

[B22] He X. Du H. Cai M. Feldman M. W. (2018). The evolution of cooperation in signed networks under the impact of structural balance. PLoS ONE 13, e0205084. 10.1371/journal.pone.0205084 30296278 PMC6175270

[B23] Hills T. T. Proto E. Sgroi D. Seresinhe C. I. (2019). Historical analysis of national subjective wellbeing using millions of digitized books. Nat. Hum. Behav. 3, 1271–1275. 10.1038/s41562-019-0750-z 31611658

[B24] Hills T. T. Todd P. M. Lazer D. Redish A. D. Couzin I. D. Group C. S. R.. (2015). Exploration versus exploitation in space, mind, and society. Trends Cogn. Sci. 19, 46–54. 10.1016/j.tics.2014.10.004 25487706 PMC4410143

[B25] Kenett Y. N. Levi E. Anaki D. Faust M. (2017). The semantic distance task: quantifying semantic distance with semantic network path length. J. Exp. Psychol. 43, 1470. 10.1037/xlm0000391 28240936

[B26] Kumar A. A. Balota D. A. Steyvers M. (2020). Distant connectivity and multiple-step priming in large-scale semantic networks. J. Exp. Psychol. 46, 2261. 10.1037/xlm0000793 31789562

[B27] Kuppens P. Van Mechelen I. Nezlek J. B. Dossche D. Timmermans T. (2007). Individual differences in core affect variability and their relationship to personality and psychological adjustment. Emotion 7, 262. 10.1037/1528-3542.7.2.262 17516805

[B28] Li H. Xie W. Luo X. Fu R. Shi C. Ying X.. (2014). Clarifying the role of psychological pain in the risks of suicidal ideation and suicidal acts among patients with major depressive episodes. Suicide Life Threat. Behav. 44, 78–88. 10.1111/sltb.12056 24106764

[B29] Li Y. Hills T. Hertwig R. (2020a). A brief history of risk. Cognition 203, 104344. 10.1016/j.cognition.2020.104344 32526519 PMC7278655

[B30] Li Y. Hills T. T. (2021). Language patterns of outgroup prejudice. Cognition 215, 104813. 10.1016/j.cognition.2021.104813 34192608

[B31] Li Y. Masitah A. Hills T. T. (2020b). The emotional recall task: juxtaposing recall and recognition-based affect scales. J. Exp. Psychol. Learn. Mem. Cogn. 46, 1782–1794. 10.31219/osf.io/r6tvh 32352820

[B32] Lovibond P. F. Lovibond S. H. (1995). The structure of negative emotional states: comparison of the depression anxiety stress scales (dass) with the beck depression and anxiety inventories. Behav. Res. Ther. 33, 335–343. 10.1016/0005-7967(94)00075-U 7726811

[B33] Lydon-Staley D. M. Zhou D. Blevins A. S. Zurn P. Bassett D. S. (2021). Hunters, busybodies and the knowledge network building associated with deprivation curiosity. Nat. Hum. Behav. 5, 327–336. 10.1038/s41562-020-00985-7 33257879 PMC8082236

[B34] Lynn C. W. Papadopoulos L. Kahn A. E. Bassett D. S. (2020). Human information processing in complex networks. Nat. Phys. 16, 965–973. 10.1038/s41567-020-0924-7

[B35] Mehler A. Gleim R. Gaitsch R. Hemati W. Uslu T. (2020). From topic networks to distributed cognitive maps: zipfian topic universes in the area of volunteered geographic information. Complexity 2020, 4607025. 10.1155/2020/4607025

[B36] Mohammad S. M. (2016). “Sentiment analysis: detecting valence, emotions, and other affectual states from text,” in Emotion Measurement (Cambridge), 201–237.

[B37] Mohammad S. (2018). “Obtaining reliable human ratings of valence, arousal, and dominance for 20,000 english words,” in Proceedings of the 56th Annual Meeting of the Association for Computational Linguistics (Volume 1: Long Papers), 174–184.

[B38] Mohammad S. M. Turney P. D. (2013). Crowdsourcing a word-emotion association lexicon. Comput. Intell. 29, 436–465. 10.1111/j.1467-8640.2012.00460.x

[B39] Moradimanesh Z. Khosrowabadi R. Gordji M. E. Jafari G. (2021). Altered structural balance of resting-state networks in autism. Sci. Rep. 11, 1–16. 10.1038/s41598-020-80330-0 33479287 PMC7820028

[B40] Newman M. (2018). Networks. London: Oxford University Press.

[B41] Oquendo M. A. Galfalvy H. C. Choo T.-H. Kandlur R. Burke A. K. Sublette M. E.. (2020). Highly variable suicidal ideation: a phenotypic marker for stress induced suicide risk. Mol. Psychiatry 26, 5079–5086. 10.1038/s41380-020-0819-0 32576966 PMC7755748

[B42] Osgood C. E. Suci G. J. Tannenbaum P. H. (1957). The Measurement of meaning. Number 47. Champaign, IL: University of Illinois Press.

[B43] Palmier-Claus J. Taylor P. J. Gooding P. Dunn G. Lewis S. (2012). Affective variability predicts suicidal ideation in individuals at ultra-high risk of developing psychosis: an experience sampling study. Br. J. Clin. Psychol. 51, 72–83. 10.1111/j.2044-8260.2011.02013.x 22268542

[B44] Pennebaker J. W. Boyd R. L. Jordan K. Blackburn K. (2015). The Development and Psychometric Properties of LIWC2015. Austin, TX: University of Texas at Austin.

[B45] Plutchik R. (1980). Emotion. A Psychoevolutionary Synthesis. New York, NY: Harper & Row.

[B46] Rizk M. M. Choo T.-H. Galfalvy H. Biggs E. Brodsky B. S. Oquendo M. A.. (2019). Variability in suicidal ideation is associated with affective instability in suicide attempters with borderline personality disorder. Psychiatry 82, 173–178. 10.1080/00332747.2019.1600219 31013205 PMC6554039

[B47] Robinson M. D. Clore G. L. (2002). Episodic and semantic knowledge in emotional self-report: evidence for two judgment processes. J. Pers. Soc. Psychol. 83, 198–215. 10.1037/0022-3514.83.1.198 12088126

[B48] Rossetti G. Citraro S. Milli L. (2021). Conformity: a path-aware homophily measure for node-attributed networks. IEEE Intell. Syst. 36, 25–34. 10.1109/MIS.2021.3051291

[B49] Sanger S. Veach P. M. (2008). The interpersonal nature of suicide: a qualitative investigation of suicide notes. Arch. Suicide Res. 12, 352–365. 10.1080/13811110802325232 18828039

[B50] Schoene A. M. Dethlefs N. (2016). “Automatic identification of suicide notes from linguistic and sentiment features,” in Proceedings of the 10th SIGHUM Workshop on Language Technology for Cultural Heritage, Social Sciences, and Humanities, 128–133.

[B51] Scollon C. N. Diener E. Oishi S. Biswas-Diener R. (2005). An experience sampling and cross cultural in- vestigation of the relation between pleasant and unpleasant affect. Cogn. Emot. 19, 27–52. 10.1080/02699930441000076

[B52] Shneidman E. S. (1999). Conceptual contribution: the psychological pain assessment scale. Suicide Life Threat. Behav. 29, 287. 10636323

[B53] Shneidman E. S. (2004). Autopsy of a Suicidal Mind. London: Oxford University Press

[B54] Siew C. S. Wulff D. U. Beckage N. M. Kenett Y. N. (2019). Cognitive network science: a review of research on cognition through the lens of network representations, processes, and dynamics. Complexity 2019, 2108423. 10.1155/2019/2108423

[B55] Škrlj B. Martinc M. Kralj J. Lavrač N. Pollak S. (2021). tax2vec: constructing interpretable features from taxonomies for short text classification. Comput. Speech Lang. 65, 101104. 10.1016/j.csl.2020.101104

[B56] Stella M. (2022). Text-mining forma mentis networks reconstruct public perception of the stem gender gap in social media. PeerJ Comput. Sci. 210, 760–766. 10.7717/peerj-cs.295 33816946 PMC7924458

[B57] Stella M. Beckage N. M. Brede M. (2017). Multiplex lexical networks reveal patterns in early word acquisition in children. Sci. Rep. 7, 46730. 10.1038/srep46730 28436476 PMC5402256

[B58] Stella M. De Nigris S. Aloric A. Siew C. S. (2019). Forma mentis networks quantify crucial differences in stem perception between students and experts. PLoS ONE 14, e0222870. 10.1371/journal.pone.0222870 31622351 PMC6797169

[B59] Stella M. Ferrara E. De Domenico M. (2018). Bots increase exposure to negative and inflammatory content in online social systems. Proc. Natl. Acad. Sci. U.S.A. 115, 12435–12440. 10.1073/pnas.1803470115 30459270 PMC6298098

[B60] Stella M. Restocchi V. De Deyne S. (2020). #lockdown: network-enhanced emotional profiling in the time of COVID-19. Big Data Cogn. Comput. 4, 14. 10.3390/bdcc4020014

[B61] Stirman S. W. Pennebaker J. W. (2001). Word use in the poetry of suicidal and nonsuicidal poets. Psychosom Med. 64, 517–522. 10.1097/00006842-200107000-00001 11485104

[B62] Teixeira A. S. Talaga S. Swanson T. J. Stella M. (2021). Revealing semantic and emotional structure of suicide notes with cognitive network science. Sci. Rep. 11, 1–15. 10.1038/s41598-021-98147-w 34593826 PMC8484592

[B63] Van Rensbergen B. De Deyne S. Storms G. (2016). Estimating affective word covariates using word association data. Behav. Res. Methods 48, 1644–1652. 10.3758/s13428-015-0680-2 26511372

[B64] Van Rensbergen B. Storms G. De Deyne S. (2015). Examining assortativity in the mental lexicon: evidence from word associations. Psychon. Bull. Rev. 22, 1717–1724. 10.3758/s13423-015-0832-5 25893712

[B65] Vitevitch M. (2019). “Can network science connect mind, brain, and behavior,” in Network Science in Cognitive Psychology (London: Routledge), 184.

[B66] Xie W. Li H. Luo X. Fu R. Ying X. Wang N.. (2014). Anhedonia and pain avoidance in the suicidal mind: behavioral evidence for motivational manifestations of suicidal ideation in patients with major depressive disorder. J. Clin. Psychol. 70, 681–692. 10.1002/jclp.22055 24307489

